# Off pump mitral valve repair

**DOI:** 10.1186/1749-8090-10-S1-A318

**Published:** 2015-12-16

**Authors:** Kestutis Rucinskas, Vilius Janusauskas, Diana Zakarkaite, Rita Kramena, Gabija Janaviciute, Agne Drasutiene, Robertas Stasys Samalavicius, Audrius Aidietis

**Affiliations:** 1 Department of Cardiovascular Disease, Vilnius University, Vilnius, Lithuania; 2 Department of Intensive Care, Centre of Anaesthesia, Intensive Care and Pain Management, Vilnius University, Vilnius, Lithuania

## Background/Introduction

The NeoChord DS1000 is a disposable device used to replace damaged chordae by delivering artificial chordae tendinae in a beating heart through small anterolateral thoracotomy. It gives alternative approach in treating degenerative mitral valve insufficiency.

## Aims/Objectives

To assess safety and efficacy in treating patients with off-pump transapical neochord implantation in Vilnius University.

## Method

60 patients had underwent off pump transapical neochord implantation. Patients were followed up to 1 year with clinical and echocardioscopic examination.

## Results

Out of 60 patients 48 (80%) had chordae implanted only to P2 segment. 2 (3%) patients had chordae implanted only to A2 segment. All other patients had chordae implanted to multiple segments. The average patient age was 60 ± 12 years, Euroscore II - 1,2 ± 1%. Median duration of operation was 129 ± 27 min. The mean intraoperative blood loss was 700 ± 500 ml, mean postoperative drainage was 230 ± 120 ml. Three (6%) patients needed RBC transfusion, 1 (2%) patient needed FFP transfusion. One patient required permanent pacemaker implantation due to sick sinus syndrome. There were no re-explorations for bleeding, strokes, new renal failure, wound infections or deaths. Acute procedure success was achieved in 58 (97%) of patients.52 patients had reached 6 months follow up and 33 patients have reached 12 months follow up. In patients with P2 prolapse without prolapse extension towards the commissures or central component to regurgitant jet or lack of central cooptation the mitral valve insufficiency less than 2+ remained in 94% (36 out of 38) patients at six months and 92 % (22 out 24) at 12 months follow up. In patients with prolapse extension towards the commissures or with central component to regurgitant jet or lack of central cooptation the mitral valve insufficiency less than 2+ was achieved in 50% (6 out of 12) patients at six months and 50% (4 out 8) at 12 months follow up.

## Discussion/Conclusion

Off-pump transapical implantation of artificial chordae with NeoChord device is a safe method for the treatment of mitral valve insufficiency and preserves conventional MV repair treatment options if unsuccessful.

